# Supplementary material for: SIDT2 Associates with Apolipoprotein A1 (ApoA1) and Facilitates ApoA1 Secretion in Hepatocytes
Source: Cells. 2023 Sep 26;12(19):2353. doi: 10.3390/cells12192353 (PMC10571540; doi:10.3390/cells12192353)

Supplemental figure 1

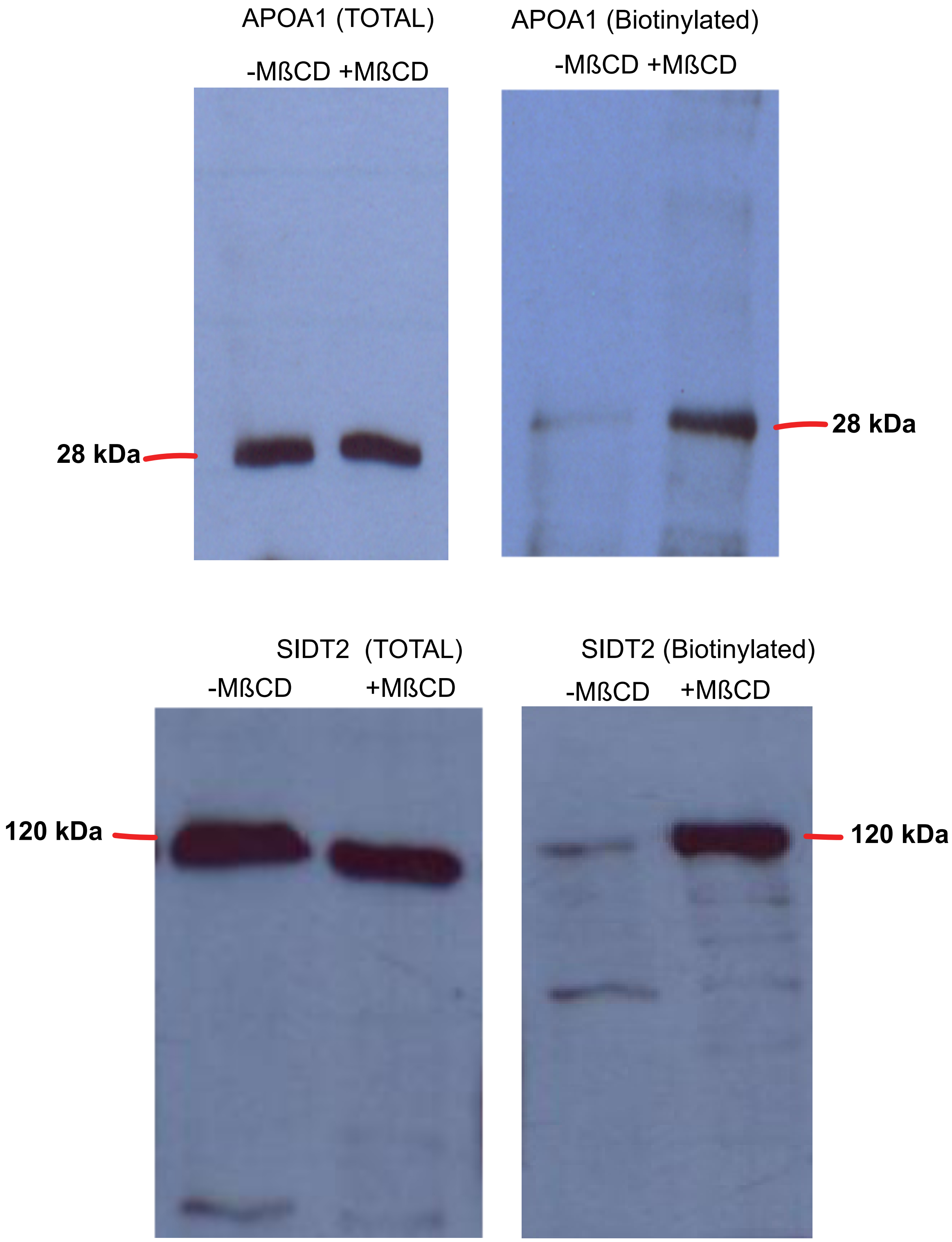

Supplemental figure 2

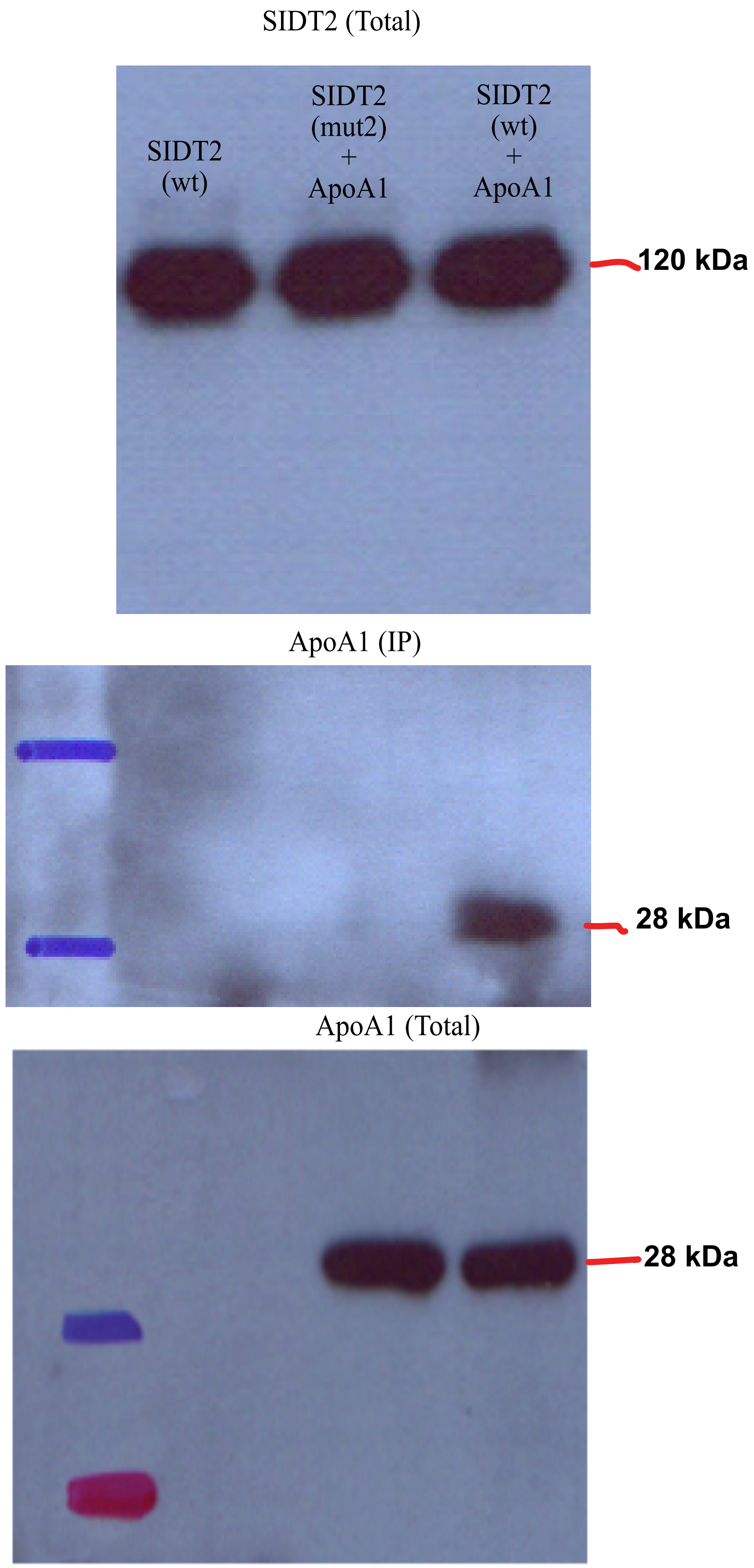

Supplemental figure 3

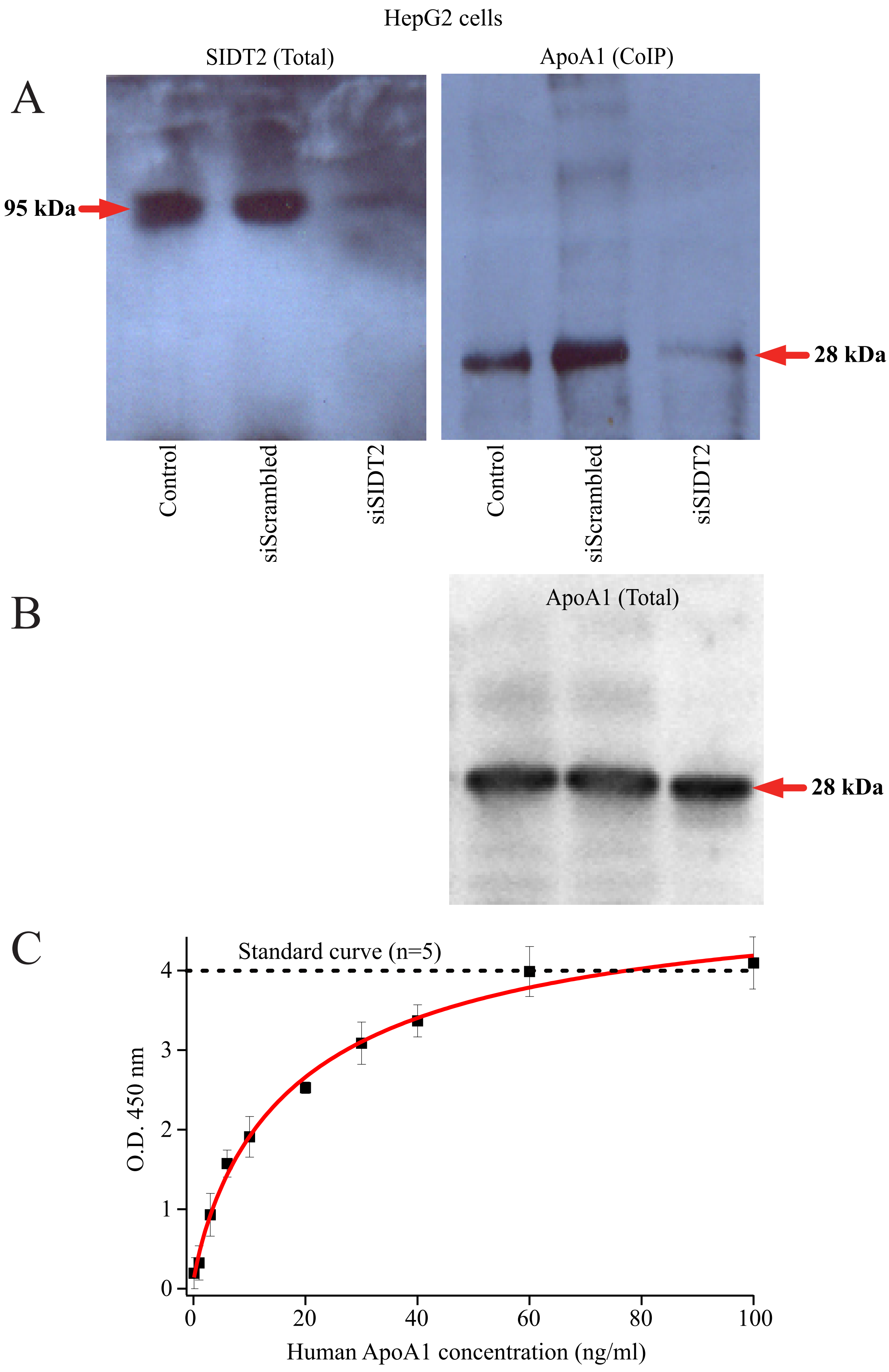

Supplement: Supplementary file 1 [file cells-12-02353-s001.zip › Supp fig 1-combined.pdf]
